# Liver X Receptor Gene Polymorphisms in Tuberculosis: Effect on Susceptibility

**DOI:** 10.1371/journal.pone.0095954

**Published:** 2014-05-01

**Authors:** Min Han, Li Liang, Li-rong Liu, Jun Yue, Yan-lin Zhao, He-ping Xiao

**Affiliations:** 1 Shanghai Key Laboratory of Mycobacterium Tuberculosis, Shanghai Pulmonary Hospital Affiliated to Tongji University School of Medicine, Shanghai, P.R. China; 2 National Center for Tuberculosis Control and Prevention, Chinese Center for Disease Control and Prevention, Beijing, P.R. China; St. Petersburg Pasteur Institute, Russian Federation

## Abstract

**Objectives:**

The Liver X receptors (LXRs), Liver X receptor A (LXRA) and Liver X receptor B (LXRB), regulate lipid metabolism and antimicrobial response. LXRs have a crucial role in the control of Mycobacterium tuberculosis (M.tb). Lacking LXRs mice is more susceptibility to infection M.tb, developing higher bacterial burdens and an increase in the size and number of granulomatous lesions. We aimed to assess the associations between single nucleotide polymorphisms (SNPs) in LXRs and risk of tuberculosis.

**Methods:**

We sequenced the LXRs genes to detect SNPs and to examine genotypic frequencies in 600 patients and 620 healthy controls to investigate for associations with tuberculosis (TB) in the Chinese Han population. DNA re-sequencing revealed eight common variants in the LXRs genes.

**Results:**

The G allele of rs1449627 and the T allele of rs1405655 demonstrated an increased risk of developing TB (p<0.001, p = 0.002), and the T allele of rs3758673, the T allele of rs2279238, and the C allele of rs1449626 in LXRA and the C allele of rs17373080, the G allele of rs2248949, and the C allele of rs1052677 in LXRB were protective against TB patients compared to healthy controls (p = 0.0002, p = 0.006, p<0.001, p = 0.004, p = 0.008, p = 0.003, respectively). All SNP genotypes were significantly associated with TB. An estimation of the frequencies of haplotypes revealed two potential risk haplotypes,GGCG in LXRB (p = 0.004,) and TTCG in LXRA (p<0.001, p = 0.004). Moreover, three protective haplotypes, TTAT and CCAT in LXRA and CATC in LXRB, were significantly “protective” (p = 0.008, p<0.001, p = 0.031) for TB. Furthermore, we determined that the LXRs SNPs were nominally associated with the clinical pattern of disease.

**Conclusions:**

Our study data supported that LXRs play a fundamental role in the genetic susceptibility to TB and to different clinical patterns of disease. Thus, further investigation is required in larger populations and in additional areas.

## Introduction

Approximately one-third of the population is infected with Mycobacterium tuberculosis (M.tb) worldwide, which is the main etiological agent of tuberculosis (TB). M.tb is a prototypic intracellular macrophage pathogen, and resides for a long period of time to avoid recognition of the human immune system during clinical dormancy. The host protective immunity plays a pivotal role in the primary immune response and active immune response, which is central in inflammation processes.

Liver X receptors (LXRs), Liver X receptor α (LXRA, NR1H3) and Liver X receptor β (LXRB, NR1H2), are a subset of nuclear receptor transcription factors that have emerged as master regulators of lipid [1], [2], inflammation [3], [4] and glucose metabolism [5], [6], [7], [8], [9]. Recently, a large body of literature has also indicated a role of LXRs in inflammation. LXRs, which are expressed in many immune cells, such as the dendritic cell [10], [11], macrophage [12], and Lymphocyte, regulate network of genes that affect many aspects of the immune system [13], [14].

In a mice study, Hannelie et al. reported that mice deficient in LXRs were more susceptible to M.tb infection, and demonstrated a decrease clearance of bacterial load in the lungs. These data support the crucial role of LXRs in the control of mycobacterial infection [13]. So we hypothesized that LXRs might also influence immunity of human. The aim of this case-controlled association study was to evaluate if LXR gene single nucleotide polymorphisms (SNPs) contribute to the susceptibility of tuberculosis in a Chinese Han population. In addition, we also investigated the role of LXR gene variants in two groups of tuberculosis subjects with different clinical patterns; pulmonary tuberculosis patients and extra- pulmonary tuberculosis patients.

## Materials and Methods

### Subjects

As a control population, we included 620 individuals (252 females, 368 males) who were older than 18 years of age and did not demonstrated inflammatory, autoimmune disease or a history of chronic infectious disease. These patients had no history of tuberculosis and no evidence of prior tuberculosis noted using chest radiography. Individuals of the control group were matched with the case group according to age, sex and ethnic.

A total of 600 patients (278 females, 322 males) were recruited from Shanghai Pulmonary Hospital in China. All patients were diagnosed on the basis of radiographic and clinical presentation, and demonstrated a positive smear and culture for M.tb. According to the different location of infection, the case subjects were then further divided into two subgroups: (1) pulmonary tuberculosis patients (PTB, n = 424) and (2) extra-pulmonary patients (EPTB, n = 176), as indicated in Table 1.

**Table 1:** Clinical characteristics of individuals stratified according to differences in infection locations.

Subgroup	Number	Male	Female	Age^c^
Control	620	368	252	37.2±8.6
PTB^a^	424	236	188	42.5±6.7
EPTB^b^	176	96	80	35.4±10.1
Lymph node tuberculosis	53	30	23	35.7±9.9
Intestine tuberculosis	8	4	4	35.4±12.9
Meningitis tuberculosis	19	11	8	37.3±11.7
Miliary tuberculosis	14	8	6	37.6±10.8
Urologic tuberculosis	5	3	2	38.8±13.3
Pleurisy tuberculosis	66	34	32	33.7±9.5
Bone tuberculosis	6	4	2	35.7±8.6
Genital tuberculosis	5	2	3	38±7.2

a PTB = pulmonary tuberculosis patients;

b EPTB = extra-pulmonary patients;

c Age (years)  = Mean ± SD.

### Ethics Statement

All controls and patients were Chinese, and all subjects were HIV-negative. Written consent was obtained from all subjects, and the Ethics Committee of the Shanghai Pulmonary Hospital Affiliated to Tongji University School of Medicine, China, provided authorization for the study.

### Screening for DNA Sequence Variants in LXR Genotyping

PCR primers were designed using Primer 3 software (http://frodo.wi.mit.edu/cgi-bin/primer3/primer3_www.cgi). The primers are shown in Table 2. To determine the frequency of known SNPs and potential functional SNPs in LXRs, the coding exons and sequence promoter regions were amplified and sequenced in 24 healthy controls and 24 patients, using previously described primers. Sequencing reactions were performed using Big-Dye v1.1 (Applied Biosystems, Foster City, USA). The sequencing data were aligned using Sequencher 4.2 (Gene Codes Corporation, Michigan, USA).

**Table 2:** Single nucleotide polymorphism (SNPs) identified using sequencing.

**SNP**	**Gene**	**Chr**	**SNP Property**	**PCR Primers** a	**Alleles**
**rs17373080**	LXRB(NR1H2)	19	5′-Flanking	F:GCAGCACGACTCCAAAATCAGA R:CGAACCCATTTCCTCGCTTCTT	G/C
**rs2248949**	LXRB(NR1H2)	19	intron 6	F:CAGTGCAACAAACGCTCCTTCT R:ACCAGGGTCACTCCCAGGTCTT	G/A
**rs1405655**	LXRB(NR1H2)	19	intron 7	F:ATGACATTCCACGGCGAATAGA R:GCCTTTTTGGCAACTTTTCTCTG	C/T
**rs1052677**	LXRB(NR1H2)	19	3′-UTR(exon 10)	F:GGTTGCAGGTCCCGACCACT R:CGCCCTCTCCATCTTGCACT	G/C
**rs3758673**	LXRA(NR1H3)	11	5′-Flanking	F:AGAAGAGGCAACCCGCATACC R:GCCTCAGCCAAGCTGGTAGAAAT	C/T
**rs2279238**	LXRA(NR1H3)	11	exon 4	F:GGGGAGAGCGTTGAAGCACTTT R:ATTTGCGAAGCCGACACTCCT	C/T
**rs1449626**	LXRA(NR1H3)	11	3′-Flanking	F:CGATCTTTAGGATCCGCCTCCA R:GGGACCGACACTGGGTTCTAGG	A/C
**rs1449627**	LXRA(NR1H3)	11	3′-Flanking	F:CGATCTTTAGGATCCGCCTCCA R:GGGACCGACACTGGGTTCTAGG	G/T

a F: forward primer, R: reverse primer.

Genomic DNA was extracted from 0.2 ml EDTA-anticoagulated blood samples using the QIAamp DNA Blood Mini Kit (Qiagen) according to the manufacturer’s protocol. Genotyping of single nucleotide polymorphisms (SNPs) was performed using the SNaPshot kit purchased from Applied Biosystems. This is a rapid and robust assay used to simultaneously genotype SNPs using single nucleotide primer extension (mini-sequencing) and can obtain population genetic data.

A 20 µl mixture was prepared to amplify all fragments in a Multiple PCR reaction and included 1x HotStarTaq buffer, 2.8 mM Mg^2+^, 0.3 mM dNTP, 0.1 µM of each primer, 1U HotStarTaq polymerase (Qiagen Inc.) and 1 µl template DNA. The cycling program was the following: 95°C for 15 min; 11 cycles of 94°C for 20 s, (62°C −0.5°C/cycle) for 40 s, 72°C for 1.5 min; 26 cycles of 94°C for 20 s, 56°C for 30 s, 72°C for 1.5 min; and 72°C for 5 min.

After the completion of multiple amplification, we used shrimp alkaline phosphatase (SAP) and ExonucleaseI (ExoI) to purify the PCR product. Next, 2U SAP and 1U ExoI were added to 5 µl of PCR product. The mixture was incubated at 37°C for 80 min, followed by incubation at 75°C for 15 min. Next, the purified PCR products were used as templates for the mini-sequencing reaction using the commercially available SNaPshot Kit. To detect polymorphisms, we used the following mini-sequencing extension primers rs2248949SF: TTTTCAGGGGCTTTGGGAAGAGG, rs3758673SF: TTTTTTTTCCCTATCCCAGGCCTGCCA, rs2279238SR: TTTAGGCCTTGTCCCCACACAC, rs1405655SF: TTTTTTTTTTTTCTGAGAAAATTGAGGATCAGGC, rs1449626SF: TTTTTTTTTTTTTTTTTTTTTTTTTTTTTTTTTGATCCGCCTCCAGGCCCAG, rs1449627SR: TTTTTTTTTTTTTTTTTTTTTTTTTTTTTTTTTTTTTTCAGATGCCAAAGGAAAA, rs17373080SF: TTTTTTTTTTTTTTTTTTTTTTTTTTTTTTTTTTTTTTTTTTTTCGCATGAATGAGCTAAAGCCA, rs1052677SF: TTTTTTTTTTTTTTTTTTTTTTTTTTTTTTTTTTTTTTTTTTTTTTTTTTTTTTATGGCTCTCCCCCCTAGCC. The SNaPshot Extension Reaction mixture included 5 µl SNaPshot mix, 2 µl purified PCR product, 2 µl ultrapure water and 2 µl extension primer mix (0.8 µM for each extension primer). The cycling protocol was the following: 96°C for 1 min; 28 cycles of 96°C for 10 s, 52°C for 5 s, and 60°C for 30 s. To purify the extension products, we added 1 U SAP to the extension products, incubated at 37°C for 60 min and then incubated at 75°C for 15 min. One microlitre of purified extension product was then mixed with 9 µl HiDi Formamide and 0.5 µl Liz120 (Applied Biosystems) size standard and denatured at 95°C for 5 min. Next, we loaded the mixture on the ABI 3730×l DNA Analyser (Applied Biosystems).

### Statistical Analysis

The allele and genotypic frequencies were determined by direct quantification. The distribution of the LXR SNPs was compared between TB patients and healthy controls using the X2 or Fisher’s exact test. The data were analyzed for appropriateness between the observed and expected genotypic values, and then evaluated for fit in Hardy-Weinberg equilibrium (HWE). HWE was calculated to ensure that all of the data were within population equilibrium. The SHEsis system (http://analysis.Bio-x.cn/SHEsisMain.htm) was used to analyze the haplotypes and determine linkage disequilibrium (LD) in the patient cases and controls. This system used a Full-Precise-Iteration (FPI) algorithm in haplotype reconstruction and frequency estimation between the patient and control [15]. The haplotypes were automatically selected. P values <0.05 were considered statistically significant.

To account for multiple comparisons, a Bonferroni correction was applied in this study so that the corrected P-values were seven times the observed P-values.

## Results

### Identification of SNPs in LXR Genes

The tagging SNPs were selected on the basis of information provided by public data-bases (http://www.ncbi.nlm.nih.gov/entrez/query.fcgi?db=snp) and re- sequencing results, using reasonable inclusion criteria. These SNPs occurred at allele frequencies of ≥0.1, according to the international Hap Map project data for Chinese populations. Three SNPS were chosen using the binning algorithm of LDSelect (http://droog.gs.washington.edu/IdSelect.pI) at γ^2^ thresholds of 0.5 and minor allele frequencies of ≥0.1.

All non- synonymous exonic variants and any variants that suggested differences in frequencies between patients and controls were included. After considering the LD structure based on Hap Map Han Chinese and pairwise LD D’ and r^2^ values, we selected the total of eight representative SNPs across the genes; four genes in LXRA and four genes in LXRB (Table 2).

### Analysis of Association between the LXR Allele and the Genotype and TB in Patients and Controls

The allele and genotypic frequencies of the eight LXR SNPs in TB patients and control subjects are shown in Table 3 and Table 4. Both populations were consistent with HWE for all polymorphisms tested (p>0.05).

**Table 3:** Allele frequencies of LXR SNPs in the TB and control groups.

SNP	Allele	Case(%)	Control(%)	x^2^	p	OR[95%CI]
**LXRA**						
rs 3758673	T	910 (75.8)	857 (69.1)	13.79	0.0002*	0.71(0.60–0.85)
	C	290 (24.2)	383 (30.9)			
rs2279238	T	814(67.8)	776(62.6)	7.14	0.006*	0.79(0.67–0.94)
	C	386(32.2)	464(37.4)			
rs1449627	G	838(69.8)	728(58.7)	32.82	<0.001*	1.62(1.38–1.92)
	T	362(30.2)	512(41.3)			
rs1449626	C	744(62.0)	665(53.6)	17.51	<0.001*	0.71(0.60–0.83)
	A	456(38.0)	575(46.4)			
**LXRB**						
rs 17373080	C	1026(85.5)	1108 (89.4)	8.26	0.004*	0.70(0.55–0.89)
	G	174 (14.5)	132 (10.6)			
rs2248949	G	1042(86.8)	1029(83.0)	7.04	0.008	0.74(0.59–0.92)
	A	158(13.2)	211 (17.0)			
rs 1405655	T	1030 (85.8)	1114(89.8)	9.18	0.002*	1.46(1.14–1.87)
	C	170 (14.2)	126(10.2)			
rs 1052677	C	1038 (86.5)	1120 (90.3)	8.72	0.003*	0.69(0.53–0.88)
	G	162(13.5)	120(9.7)			

*P indicates the significant association after Bonferroni correction for multiple testing at the significance level a = 0.05.

**Table 4:** Genotypic frequencies of LXR SNPs in the TB and control groups.

SNP	Genotype	Cases (%)	Controls (%)	x^2^	P
**LXRA**					
rs3758673	T/T	346(57.7)	279(45.0)	20.01	<0.001*
	C/T	218(36.3)	299(48.2)		
	C/C	36(6.0)	42(6.8)		
rs2279238	T/T	270(45.0)	229(36.9)	8.55	0.014
	C/T	274(45.7)	318(51.3)		
	C/C	56(9.3)	73(11.8)		
rs1449627	G/G	288(48.0)	180(29.0)	46.41	<0.001*
	G/T	262(43.7)	368(59.4)		
	T/T	50(8.3)	72(11.6)		
rs1449626	C/C	220(36.7)	154(24.8)	21.46	<0.001*
	A/C	304(50.7)	357(57.6)		
	A/A	76(12.7)	109(17.6)		
**LXRB**					
rs 17373080	C/C	444(74.0)	494(79.7)	9.60	0.008
	C/G	138(23.0)	120(19.4)		
	G/G	18(3.0)	6(1.0)		
rs2248949	G/G	452(75.3)	439(70.8)	10.45	0.005*
	A/G	138(23.0)	151(24.4)		
	A/A	10(1.7)	30(4.8)		
rs 1405655	T/T	446(74.3)	500(80.6)	9.59	0.008
	C/T	138(23.0)	114(18.4)		
	C/C	16(2.7)	6(1.0)		
rs 1052677	C/C	454(75.7)	506(81.6)	9.07	0.01
	C/G	130(21.7)	108(17.4)		
	G/G	16(2.7)	6(1.0)		

*P indicates the significant association after Bonferroni correction for multiple testing at the significance level a = 0.05.

Analyses of the contribution of LXR gene allele frequencies revealed that the G allele of rs1449627 in LXRA and the T allele of rs1405655 in LXRB were more common in TB patients compared healthy controls (p<0.001, OR (95%CI)  = 1.62(1.38–1.92); p = 0.002, OR (95%CI)  = 1.46 (1.14–1.87), respectively); the T allele of rs3758673, T allele of rs2279238, and C allele of rs1449626 in LXRA and the C allele of rs17373080, G allele of rs2248949, C allele of rs1052677 in LXRB were less in TB patients compared to healthy controls (p = 0.0002, OR (95%CI) = 0.71(0.60–0.85), p = 0.006, OR (95%CI)  = 0.79(0.67–0.94), p<0.001, OR (95%CI)  = 0.71(0.60–0.83), p = 0.004, OR (95%CI)  = 0.70(0.55–0.89), p = 0.008, OR (95%CI)  = 0.74(0.59–0.92), p = 0.003, OR (95%CI)  = 0.69(0.53–0.88), respectively).

Analysis of LXRs genotype frequencies demonstrated that the rs3758673 T/T, rs2279238 T/T, rs1449627 G/G, and rs1449626 C/C in LXRA were significantly more common in TB patients than healthy controls (p<0.001, p = 0.014, p<0.001 and p = <0.001, respectively). In the case of LXRB gene, rs17373080 C/C, rs1052677 C/C, rs2248949 G/G and rs1405655 T/T genotype variants were associated with TB (p = 0.008, p = 0.005, p = 0.008 and p = 0.01, respectively).

Statistical significant differences in the allele (G allele of rs2248949), and genotypes (rs2279238 of LXRA, and rs17373080, rs1405655, rs1052677 of LXRB), however, were lost after Bonferroni correction of the p values (Pc).

### Linkage Disequilibrium (LD) and Haplotype Analyses

**Figure 1:**
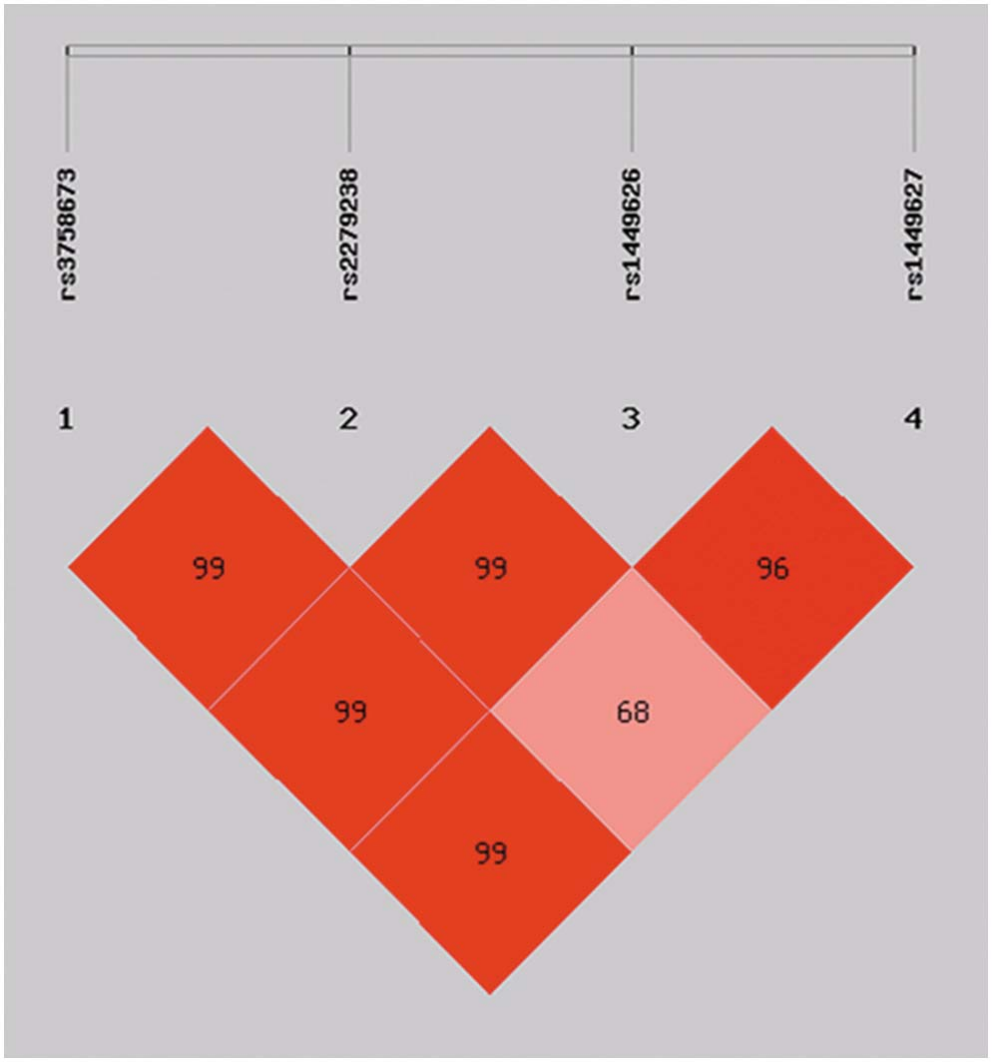
Linkage disequilibrium (LD) structure of the SNPs in LXRA. The D’ values (%) are indicated on the squares. Pairwise D’ values are colour coded: high D’ values are dark, low D’ values are light. All D’ values were generated by SHEsis software.

**Figure 2:**
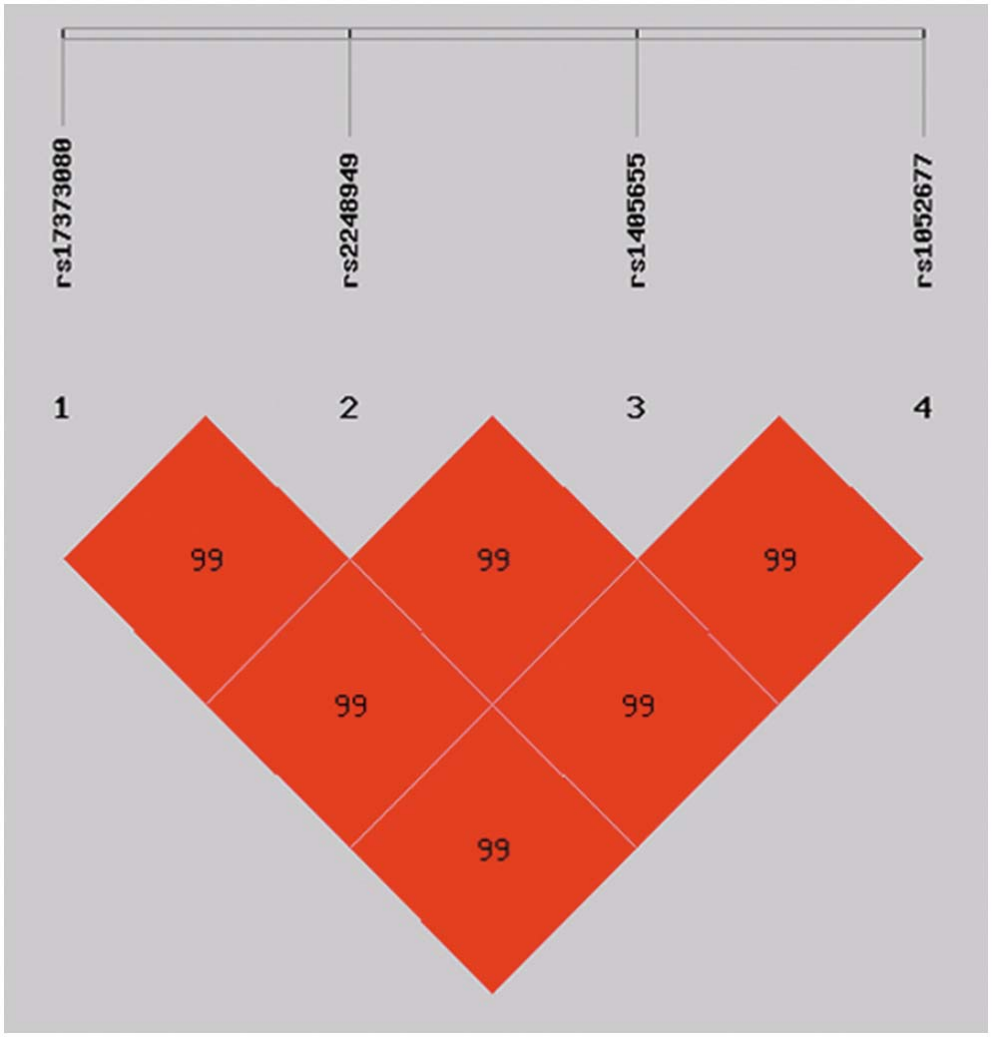
Linkage disequilibrium (LD) structure of the SNPs in LXRB. The D’ values (%) are indicated on the squares. Pairwise D’ values are colour coded: high D’ values are dark, low D’ values are light. All D’ values were generated by SHEsis software.

The LD pattern across SNPs was assessed using both D’ and r^2^, and the LD structure (LXRA and LXRB) was shown in Fig. 1 and Fig. 2. Thus, the haplotypes were constructed with all eight variants and the frequencies of the haplotypes were analyzed by excluding the rare haplotypes (those with a frequency <0.03 in cases or controls). This revealed a significant difference in the distribution of the global haplotypes between the TB group and healthy controls (p = 0.001). SHEsis identified four haplotypes for markers genotyped in LXRA and two haplotypes for makers in LXRB (Table 5). The degree of LD over the genomic region containing LXRs was high. Moreover, the respective gene generated one haploblock (Fig. 1 and Fig. 2).

**Table 5:** Estimated frequencies of haplotypes consisting of LXR SNPs in cases and controls.

Haplotype	Case(%)*	Control(%)*	x^2^	p-value	OR(95%CI)
**LXRB**					
CATC	155.21(0.129)	198.91(0.16)	4.664	0.031	0.779(0.621–0.978)
CGTC	870.79(0.726)	909.09(0.733)	0.115	0.735	0.969(0.808–1.162)
GGCG	159.90(0.133)	119.91(0.097)	8.139	0.004*	1.440(1.120–1.852)
Global	1240	1200	11.21	0.004	
**LXRA**					
CCAT	290.00(0.242)	383.00(0.309)	16.602	<0.001*	0.689(0.576–0.825)
TCAG	94.00(0.078)	77.41(0.062)	1.908	0.167	1.245(0.912–1.702)
TTAT	70.00(0.058)	104.87(0.085)	7.134	0.008	0.654(0.478–0.895)
TTCG	744.00(0.58)	644.46(0.520)	19.228	<0.001*	1.437(1.222–1.691)
Global	1240	1200	28.49	<0.001	

*Frequencies <0.03 were excluded from the analysis.

*P indicates the significant association after Bonferroni correction for multiple testing at the significance level a = 0.05.

The most common haplotypes in healthy controls and TB patients were TTCG in LXRA and GGCG in LXRB. Haplotypes analysis revealed two haplotypes in LXRB (CATC and GGCG) and three haplotypes in LXRA (CCAT, TTAT and TTCG), which were significantly associated with TB. The GGCG haplotype and TTCG haplotype was a significant “at-risk” haplotype (p = 0.004, OR (95%CI)  = 1.440 (1.120–1.852); p<0.001, OR (95%CI)  = 1.437 (1.222–1.691)). The TTAT and CCAT in LXRA and CATC in LXRB was a significant “protective” haplotype (p = 0.008, OR (95%CI)  = 0.654 (0.478–0.895), P<0.001, OR (95%CI)  = 0.689 (0.576–0.825); P  = 0.031, OR (95%CI)  = 0.779 (0.621–0.978)). Only the relationship between TTCG and CCAT in LXRA, and GGCG in LXRB and TB were still significant after Bonferroni correction.

### Comparison of LXR Data between Pulmonary TB Patients and Extra-pulmonary TB Patients

To further understand the association between LXR SNPs and the risk of different clinical patterns, we evaluated the SNP contribution on clinical variables related to tuberculosis. No significant association was detected between the allele of LXRs and the risk of different clinical forms of tuberculosis (date not shown). However, the genotype at the LXRA SNP rs1449627 and LXRB SNPs rs17373080, rs1405655 and rs1052677 was associated nominally associated to PTB, p = 0.006, p = 0.013, p = 0.004 and p = 0.0007, respectively. We also performed haplotype analyses using the LXR SNPs in two clinical groups. Seven haplotypes were observed in samples of two subgroups, but only the CATC haplotype in LXRB demonstrated a significant association with an increase in the risk of PTB (p = 0.045 OR (95%CI)  = 1.511 (1.007–2.269)). After Bonferroni correction, CATC haplotype and LXRB SNPs rs17373080 were lost significant associated with the different clinical forms of the disease.

## Discussion

Tuberculosis, a chronic inflammatory disease that occurs within the entire body, is a complex process, and can cause multi-system complications, such as pulmonary, osteoarticular, and renal, central nerve systems (CNS). The initiation and progression of tuberculosis involves a dynamic interplay between numerous signaling pathways and cell types, where macrophages, dendritic cells (DCs), T cells, and other cellular elements present in the infection sites contribute to its pathogenesis. Many studies have investigated the potential role of LXRs in various bacterial infections, such as *Listeria monocytogenes* [16], *Leishmania* [17], *Mycobacterium tuberculosis* [13]. However, in different infections, the phenotypic effect is more discriminatory, in which a multiplicity host-association is present between the pathogen and host immune system.

We have analyzed eight common genetic variations in LXRs, LXRA and LXRB, for an association with tuberculosis in a Chinese Han population and for an association with different disease clinical patterns in two subgroups of tuberculosis patients. We assessed the hypothesis that sequence variations in the LXR gene may contribute to TB susceptibility. We screened LXR genes for sequence variations in 24 healthy controls and 24 TB patients, resulting in eight representative SNPs across genes, four SNPs for each respective gene.

In this study, we observed an association of LXR SNPs with TB. Frequencies of the allele and genotype in the LXR genes were different between the healthy controls and TB patients. The G allele of rs1449627 in LXRA and the T allele of rs1405655 in LXRB were more common in TB patients compare to healthy controls. The T allele of rs3758673, the T allele of rs2279238, and the C allele of rs1449626 in LXRA and the C allele of rs17373080, the G allele of rs2248949, the C allele of rs1052677 in LXRB exhibited a significant protective effect. Moreover, all genotypes of LXRs were significantly associated with TB. Statistical significant differences in the allele (G allele of rs2248949), and genotypes (rs2279238 of LXRA, and rs17373080, rs1405655, rs1052677 of LXRB), however, were lost after Bonferroni correction of the p values (Pc).

To investigate a potential role of LXR SNPs in two subgroups of TB patients, we evaluated the SNP contribution on the clinical variables related to tuberculosis. No LXR allele associated to the investigated phenotypes in two patient subgroups was observed. In addition, the genotype at the LXRA SNP rs1449627 and LXRB SNPs s17373080, rs1405655, and rs1052677 was nominally associated to pulmonary TB, and the haplotype CATC in LXRB was significantly associated with an increased risk of PTB. After Bonferroni correction, CATC haplotype and LXRB SNPs rs17373080 were lost significant associated with disease risk.

The LXRB SNP rs17373080 in the promoter region was nominally associated with TB, particularly with PTB. A previously study revealed that the LXRB promoter SNP rs17373080, resides proximally to the transcription start site and could potentially contribute to the transcription potential of the LXRB gene promoter [18]. The strong LD over the genomic region containing LXRB may underlie the intronic SNP association between rs2248949 and rs1405655 with disease. Thus, further studies investigating the effect of various alleles at this position in relationship to the LXRB promoter activity and transcription factor binding are needed. The LXRA SNP rs2279238 is located at an exon splicing enhancer (ESE) where splicing factor SRp55 binds but the nucleotide variation is synonymous, resulting in a protein sequence identical to that of the wild type [19].

The LXRs, LXRA and LXRB, are ligand-activated transcription factors that belong to the nuclear hormone receptor super-family, and have emerged as important regulators of lipid metabolism and inflammation. LXRA is highly expressed in liver, and at lower levels in the intestine, macrophages, adipose tissue, lung, adrenal glands and kidney, all of which play important roles in lipid homeostasis. In contract, LXRB is ubiquitously expressed [20]. Activation of the LXRs inhibited inflammatory gene expression of inflammatory cytokines, including TLR4, IL-1β, and TNF-α [21], [22]. In recent years, there has been growing evidence that LXRA and LXRB exert key inhibitory roles in the control of inflammation [4]. Moreover, many studies have reported a crucial role for LXR in inhibiting inflammation in models of atherosclerosis [23], colorectal cancer [24], lung inflammation [25], [26], autoimmune encephalomyelitis [27], spinal cord trauma [28], and sepsis [29]. Modulation of inflammatory signaling by LXRs substantiates its use as a therapeutic target for inflammatory diseases. With respect to pathological bone disease, activation of LXRs has been demonstrated to play a protective role in the prevention of rheumatoid arthritis [30], osteoarthritis [31], [32], and postmenopausal osteoporosis [33]. In the past years, there have been several experiments focusing on the association between LXRA and/or LXRB gene polymorphisms with different diseases. Dahlman et al. reported that LXRA SNP rs2279238 CC carriers had lower body mass index and rs2279238 CT carriers were associated with obesity phenotypes [34], [35]. Legry et al. documented that rs11039155 AA carriers of LXRA had higher HDL cholesterol and a 30% decrease in risk of having the metabolic syndrome in two cohorts of French subjects [36]. In contract, Rooki et al. have documented that two common SNPs in LXRA (rs11039155 and rs2279238) and in LXRB (rs17373080 and rs2695121) are not potential contributors to the risk of metabolic syndrome and related traits in an Iranian population [37].

In conclusion, our results suggested associations between common genetic variants of LXRs and the risks of pulmonary tuberculosis, confirming the recent study by Korf H et al. This investigation suggested that gene polymorphisms in LXRs are likely to affect the risk of developing tuberculosis, and demonstrated the potential of LXR modulators as therapeutic targets for tuberculosis. However, these findings must be interpreted in other large population samples and performing meta-analyses are necessary before a link between LXRs gene variability and tuberculosis can definitely be established. Moreover, understanding the molecular mechanisms behind the LXRs SNP-disorder association will require further experimental research.

## References

[1] Ross SE, Erickson RL, Gerin I, DeRose PM, Bajnok L, et al (2002) Microarray analyses during adipogenesis: understanding the effects of Wnt signaling on adipogenesis and the roles of liver X receptor alpha in adipocyte metabolism. Mol Cell Biol 22: 5989–5999. 12138207 10.1128/MCB.22.16.5989-5999.2002 PMC133961

[2] Ricote M, Valledor AF, Glass CK (2004) Decoding transcriptional programs regulated by PPARs and LXRs in the macrophage: effects on lipid homeostasis, inflammation, and atherosclerosis. Arterioscler Thromb Vasc Biol 24: 230–239. 14592855 10.1161/01.ATV.0000103951.67680.B1

[3] Valledor AF (2005) The innate immune response under the control of the LXR pathway. Immunobiology 210: 127–132. 16164019 10.1016/j.imbio.2005.05.007

[4] Treuter E, Venteclef N (2011) Transcriptional control of metabolic and inflammatory pathways by nuclear receptor SUMOylation. Biochim Biophys Acta 1812: 909–918. 21172431 10.1016/j.bbadis.2010.12.008

[5] Steffensen KR, Gustafsson JA (2004) Putative metabolic effects of the liver X receptor (LXR) Diabetes. 53 Suppl 1S36–42. 10.2337/diabetes.53.2007.s36 14749264

[6] Cao G, Liang Y, Broderick CL, Oldham BA, Beyer TP, et al (2003) Antidiabetic action of a liver x receptor agonist mediated by inhibition of hepatic gluconeogenesis. J Biol Chem 278: 1131–1136. 12414791 10.1074/jbc.M210208200

[7] Laffitte BA, Chao LC, Li J, Walczak R, Hummasti S, et al (2003) Activation of liver X receptor improves glucose tolerance through coordinate regulation of glucose metabolism in liver and adipose tissue. Proc Natl Acad Sci USA 100: 5419–5424. 12697904 10.1073/pnas.0830671100 PMC154360

[8] Anthonisen EH, Berven L, Holm S, Nygård M, Nebb HI, et al (2010) Nuclear receptor liver X receptor is O-GlcNAc-modified in response to glucose. J Biol Chem 285: 1607–1615. 19933273 10.1074/jbc.M109.082685 PMC2804318

[9] Mitro N, Mak PA, Vargas L, Godio C, Hampton E, et al (2007) The nuclear receptor LXR is a glucose sensor. Nature 445: 219–223. 17187055 10.1038/nature05449

[10] Töröcsik D, Baráth M, Benko S, Széles L, Dezso B, et al (2010) Activation of liver X receptor sensitizes human dendritic cells to inflammatory stimuli. J Immunol 184: 5456–5465. 20410489 10.4049/jimmunol.0902399

[11] Geyeregger R, Zeyda M, Bauer W, Kriehuber E, Säemann MD, et al (2007) Liver X receptors regulate dendritic cell phenotype and function through blocked induction of the actin-bundling protein fascin. Blood 109: 4288–4295. 17255360 10.1182/blood-2006-08-043422

[12] Joseph SB, Bradley MN, Castrillo A, Bruhn KW, Mak PA, et al (2004) LXR-dependent gene expression is important for macrophage survival and the innate immune response. Cell 119: 299–309. 15479645 10.1016/j.cell.2004.09.032

[13] Korf H, Vander Beken S, Romano M, Steffensen KR, Stijlemans B, et al (2009) Liver X receptors contribute to the protective immune response against Mycobacterium tuberculosis in mice. J Clin Invest 119: 1626–1637. 19436111 10.1172/JCI35288 PMC2689129

[14] Bensinger SJ, Bradley MN, Joseph SB, Zelcer N, Janssen EM, et al (2008) LXR signaling couples sterol metabolism to proliferation in the acquired immune response. Cell 134: 97–111. 18614014 10.1016/j.cell.2008.04.052 PMC2626438

[15] Shi YY, He L (2005) SHEsis, a powerful software platform for analyses of linkage disequilibrium, haplotype construction, and genetic association at polymorphism loci. Cell Res 15: 97–98. 15740637 10.1038/sj.cr.7290272

[16] Joseph SB, McKilligin E, Pei L, Watson MA, Collins AR, et al (2002) Synthetic LXR ligand inhibits the development of atherosclerosis in mice. Proc Natl Acad Sci USA 99: 7604–7609. 12032330 10.1073/pnas.112059299 PMC124297

[17] Bruhn KW, Marathe C, Maretti-Mira AC, Nguyen H, Haskell J, et al (2010) LXR deficiency confers increased protection against visceral Leishmania infection in mice. PLoS Negl Trop Dis 4: e886. 21103366 10.1371/journal.pntd.0000886 PMC2982826

[18] Dahlman I, Nilsson M, Gu HF, Lecoeur C, Efendic S, et al (2009) Functional and genetic analysis in type 2 diabetes of liver X receptor alleles–a cohort study. BMC Med Genet 10: 27. 19292929 10.1186/1471-2350-10-27 PMC2664799

[19] Price ET, Pacanowski MA, Martin MA, Cooper-DeHoff RM, Pepine CJ, et al (2011) Liver X receptor α gene polymorphisms and variable cardiovascular outcomes in patients treated with antihypertensive therapy: results from the INVEST-GENES study. Pharmacogenet Genomics 21: 333–340. 21562465 10.1097/FPC.0b013e3283452fec PMC3093636

[20] Repa JJ, Mangelsdorf DJ (2000) The role of orphan nuclear receptors in the regulation of cholesterol homeostasis. Annu Rev Cell Dev Biol 16: 459–481. 11031244 10.1146/annurev.cellbio.16.1.459

[21] Joseph SB, Castrillo A, Laffitte BA, Mangelsdorf DJ, Tontonoz P (2003) Reciprocal regulation of inflammation and lipid metabolism by liver X receptors. Nat Med 9: 213–219. 12524534 10.1038/nm820

[22] Fowler AJ, Sheu MY, Schmuth M, Kao J, Fluhr JW, et al (2003) Liver X receptor activators display anti-inflammatory activity in irritant and allergic contact dermatitis models: liver-X-receptor-specific inhibition of inflammation and primary cytokine production. J Invest Dermatol 120: 246–255. 12542530 10.1046/j.1523-1747.2003.12033.x

[23] Calkin AC, Tontonoz P (2010) Liver x receptor signaling pathways and atherosclerosis. Arterioscler Thromb Vasc Biol 30: 1513–1518. 20631351 10.1161/ATVBAHA.109.191197 PMC2919217

[24] Lo Sasso G, Bovenga F, Murzilli S, Salvatore L, Di Tullio G, et al (2013) Liver X receptors inhibit proliferation of human colorectal cancer cells and growth of intestinal tumors in mice. Gastroenterology 144: 1497–1507 1507: e1–13. 10.1053/j.gastro.2013.02.005 23419360

[25] Smoak K, Madenspacher J, Jeyaseelan S, Williams B, Dixon D, et al (2008) Effects of liver X receptor agonist treatment on pulmonary inflammation and host defense. J Immunol 180: 3305–3312. 18292555 10.4049/jimmunol.180.5.3305 PMC2430066

[26] Birrell MA, Catley MC, Hardaker E, Wong S, Willson TM, et al (2007) Novel role for the liver X nuclear receptor in the suppression of lung inflammatory responses. J Biol Chem 282: 31882–31890. 17766241 10.1074/jbc.M703278200

[27] Hindinger C, Hinton DR, Kirwin SJ, Atkinson RD, Burnett ME, et al (2006) Liver X receptor activation decreases the severity of experimental autoimmune encephalomyelitis. J Neurosci Res 84: 1225–1234. 16955483 10.1002/jnr.21038

[28] Paterniti I, Genovese T, Mazzon E, Crisafulli C, Di Paola R, et al (2010) Liver X receptor agonist treatment regulates inflammatory response after spinal cord trauma. J Neurochem 112: 611–624. 19891733 10.1111/j.1471-4159.2009.06471.x

[29] Wang YY, Ryg U, Dahle MK, Steffensen KR, Thiemermann C, et al (2011) Liver X receptor protects against liver injury in sepsis caused by rodent cecal ligation and puncture. Surg Infect (Larchmt) 12: 283–289. 21815813 10.1089/sur.2010.066

[30] Park MC, Kwon YJ, Chung SJ, Park YB, Lee SK (2010) Liver X receptor agonist prevents the evolution of collagen-induced arthritis in mice. Rheumatology (Oxford) 49: 882–890. 20159908 10.1093/rheumatology/keq007

[31] Tsezou A, Iliopoulos D, Malizos KN, Simopoulou T (2010) Impaired expression of genes regulating cholesterol efflux in human osteoarthritic chondrocytes. J Orthop Res 28: 1033–1039. 20108316 10.1002/jor.21084

[32] Collins-Racie LA, Yang Z, Arai M, Li N, Majumdar MK, et al (2009) Global analysis of nuclear receptor expression and dysregulation in human osteoarthritic articular cartilage: reduced LXR signaling contributes to catabolic metabolism typical of osteoarthritis. Osteoarthritis Cartilage 17: 832–842. 19217805 10.1016/j.joca.2008.12.011

[33] Kleyer A, Scholtysek C, Bottesch E, Hillienhof U, Beyer C, et al (2012) Liver X receptors orchestrate osteoblast/osteoclast crosstalk and counteract pathologic bone loss. J Bone Miner Res 27: 2442–2451. 22806960 10.1002/jbmr.1702

[34] Dahlman I, Nilsson M, Jiao H, Hoffstedt J, Lindgren CM, et al (2006) Liver X receptor gene polymorphisms and adipose tissue expression levels in obesity. Pharmacogenet Genomics 16: 881–889. 17108812 10.1097/01.fpc.0000236334.49422.48

[35] Dahlman I, Nilsson M, Gu HF, Lecoeur C, Efendic S, et al (2009) Functional and genetic analysis in type 2 diabetes of liver X receptor alleles–a cohort study. BMC Med Genet 10: 27. 19292929 10.1186/1471-2350-10-27 PMC2664799

[36] Legry V, Cottel D, Ferrières J, Chinetti G, Deroide T, et al (2008) Association between liver X receptor alpha gene polymorphisms and risk of metabolic syndrome in French populations. Int J Obes (Lond) 32: 421–428. 18209740 10.1038/sj.ijo.0803705

[37] Rooki H, Ghayour-Mobarhan M, Haerian MS, Ebrahimi M, Azimzadeh P, et al (2013) Lack of association between LXRα and LXRβ gene polymorphisms and prevalence of metabolic syndrome: a case-control study of an Iranian population. Gene 532: 288–293. 24100084 10.1016/j.gene.2013.09.107

